# Role of Myotonic Dystrophy Protein Kinase (DMPK) in Glucose Homeostasis and Muscle Insulin Action

**DOI:** 10.1371/journal.pone.0001134

**Published:** 2007-11-07

**Authors:** Esther Llagostera, Daniele Catalucci, Luc Marti, Marc Liesa, Marta Camps, Theodore P. Ciaraldi, Richard Kondo, Sita Reddy, Wolfgang H. Dillmann, Manuel Palacin, Antonio Zorzano, Pilar Ruiz-Lozano, Ramon Gomis, Perla Kaliman

**Affiliations:** 1 Departament de Bioquímica i Biologia Molecular, Facultat de Biologia, Universitat de Barcelona, Barcelona, Spain; 2 University of California at San Diego, La Jolla, California, United States of America; 3 Istituto per la Ricerca e Cura del Cancro (IRCC) Multimedica, Milan, Italy; 4 Institute for Research in Biomedicine, Barcelona Science Park, Barcelona, Spain; 5 Institute for Genetic Medicine, University of Southern California at Los Angeles, Los Angeles, California, United States of America; 6 The Burnham Institute for Medical Research, La Jolla, California, United States of America; 7 Endocrinology and Diabetes Unit, Institut d'Investigacions Biomèdiques August Pi i Sunyer (IDIBAPS), Hospital Clinic de Barcelona, Barcelona, Spain; Columbia University, United States of America

## Abstract

Myotonic dystrophy 1 (DM1) is caused by a CTG expansion in the 3′-unstranslated region of the DMPK gene, which encodes a serine/threonine protein kinase. One of the common clinical features of DM1 patients is insulin resistance, which has been associated with a pathogenic effect of the repeat expansions. Here we show that DMPK itself is a positive modulator of insulin action. DMPK-deficient (*dmpk* ^−/−^) mice exhibit impaired insulin signaling in muscle tissues but not in adipocytes and liver, tissues in which DMPK is not expressed. *Dmpk* ^−/−^ mice display metabolic derangements such as abnormal glucose tolerance, reduced glucose uptake and impaired insulin-dependent GLUT4 trafficking in muscle. Using DMPK mutants, we show that DMPK is required for a correct intracellular trafficking of insulin and IGF-1 receptors, providing a mechanism to explain the molecular and metabolic phenotype of *dmpk* ^−/−^ mice. Taken together, these findings indicate that reduced DMPK expression may directly influence the onset of insulin-resistance in DM1 patients and point to *dmpk* as a new candidate gene for susceptibility to type 2-diabetes.

## Introduction

Type 2 diabetes is a heterogeneous disease and a major international public health threat. It is widely accepted that type 2 diabetes results from a combination of genetic susceptibility and other risk factors including obesity, increased age, hypertension, and lifestyle [1]. Insulin resistance, which is a major factor in the development of type 2 diabetes [2], is a common metabolic feature in myotonic dystrophy 1 (DM1), an autosomal dominant neuromuscular disorder [3]. DM1 patients frequently exhibit normal basal insulin levels but excessive insulin release after a glucose load, indicating a compensatory beta-cell response to tissue insulin insensitivity [4], [5]. Whole-body glucose disposal in DM1 patients is reduced by 15−25% following insulin infusion [6] and experiments with forearm muscle indicate a 70% decrease in insulin sensitivity in skeletal muscle [7]. The DM1 mutation has been identified as the expansion of an unstable CTG-repeat in the 3′-untranslated region of a gene encoding DMPK (myotonic dystrophy protein kinase) [8], [9]. Insulin resistance in DM1 has been associated with aberrant splicing of the insulin receptor RNA due to a toxic effect of the CUG-expanded repeats, which are transcribed from the mutated *dmpk* gene but are retained in the nucleus altering the normal metabolism of RNAs [10], [11]. However, whether the entire endocrine pathology of DM1 is caused by alterations in RNA processing remains to be seen. Indeed, DM1 patients show a 50% decrease in DMPK expression [12] and studies of *dmpk* knockout mice indicate that at least some of the features of DM1 result from haploinsufficiency of DMPK [13]–[15]. Interestingly, *dmpk* gene is located on chromosome 19q13, in which quantitative trait loci (QTLs) for type 2 diabetes-associated phenotypes have been identified by two independent genome-wide linkage scans among large and multiple ethnicity populations [16], [17].

DMPK is mainly expressed in muscle [18], which is a key target tissue for insulin-dependent regulation of glucose metabolism [19]. Structurally, DMPK presents homology with protein kinases of the Rho family (Rho-kinase), which have important roles in the organization of the cytoskeleton and several cellular processes including intracellular protein trafficking and metabolism [20], [21]. Although little is known about the mechanisms that regulate DMPK activity, it has been described that DMPK is activated in response to G protein second messengers [22] and that the actin cytoskeleton-linked GTPase Rac-1 binds to DMPK, promoting its transphosphorylation activity in a GTP-sensitive manner [23]. Here we examined the role of DMPK in the regulation of insulin action and glucose homeostasis using a DMPK-deficient mouse model [13]. We show that DMPK plays a role in the regulation of whole-body glucose disposal and muscle insulin sensitivity through a mechanism that involves the intracellular trafficking of insulin and IGF-1 receptors.

## Results

### *Dmpk* ^−/−^ mice exhibit insulin signaling defects in skeletal and cardiac muscles

**Figure 1:**
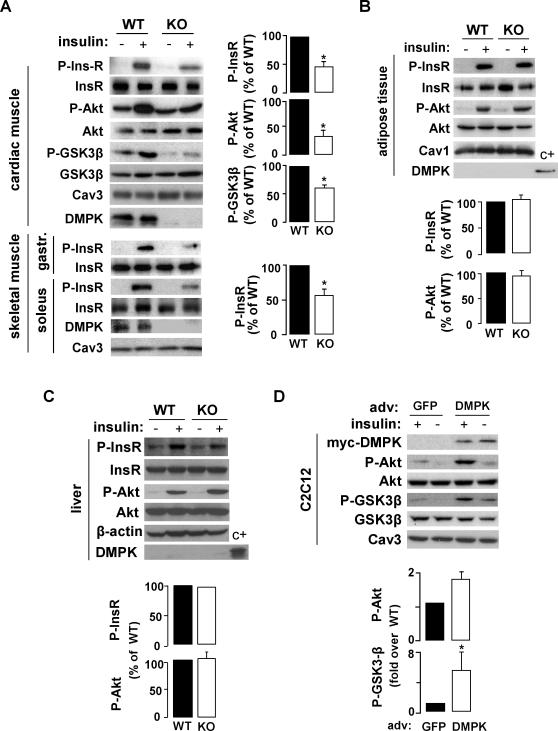
DMPK regulates muscle insulin signaling. (A) Cardiac and skeletal muscles (soleus and gastrocnemius); (B) white adipose tissue; and (C) liver homogenates from *dmpk* ^−/−^ and wild type mice were analysed by Western blot. Caveolin 3, caveolin 1 and β-actin were used as loading controls. Heart homogenate was loaded as positive control for DMPK expression in B and C. (D) C2C12 myoblasts transduced with recombinant adenovirus for myc-DMPK or green fluorescent protein (GFP) as control were analyzed by Western blot. Caveolin 3 was used as loading control. Data are means±SEM. *P<0.05 vs control values (n = 3-5).

We used *dmpk*–null mice to explore the involvement of DMPK in insulin action. We first analyzed cardiac and skeletal muscles, in which DMPK is preferentially expressed [18]. *Dmpk* ^−/−^ mice showed normal expression of the insulin receptor and other components of the insulin signaling pathway, such as the protein kinase Akt and glycogen synthase kinase 3β (GSK3-β) (Fig. 1 A). However, abnormalities were found in the activation of the insulin signaling pathway in both cardiac and skeletal muscle. Insulin-induced autophosphorylation of the insulin/IGF-I receptor (Tyr1150/1151-InsR; Tyr1135/1136-IGF-1R) was substantially decreased in *dmpk* ^−/−^ mice after insulin treatment *in vivo* (54±10% decrease in cardiac muscle; 44±10% decrease in skeletal muscle) (Fig. 1 A). Phosphorylation of other components of the insulin signaling pathway such as Ser473-Akt and Ser9-GSK3-β was also severely reduced in cardiac muscle from *dmpk* ^−/−^ mice (39±5% decrease in Akt phosphorylation; 67±9% decrease in GSK3-β phosphorylation) (Fig. 1 A). In contrast, insulin-induced autophosphorylation of the insulin/IGF-I receptor and phosphorylation of Ser473-Akt were preserved in adipose tissue and liver (Fig. 1 B and C, respectively), in which DMPK is not expressed. These results suggest that insulin signaling defects in *dmpk* ^−/−^ mice are restricted to DMPK-expressing tissues. A role of DMPK in muscle insulin signaling was corroborated by overexpression of DMPK in C2C12 skeletal muscle cells. In myoblasts transduced with myc-tagged wild-type DMPK-adenovirus, insulin-stimulated phosphorylation of Ser473-Akt and Ser9-GSK3-β was increased 1.8±0.2- and 5.5±2.6-fold, respectively, compared to control cells (Fig. 1 D).

### Metabolic alterations in *dmpk* ^−/−^ mice

**Figure 2:**
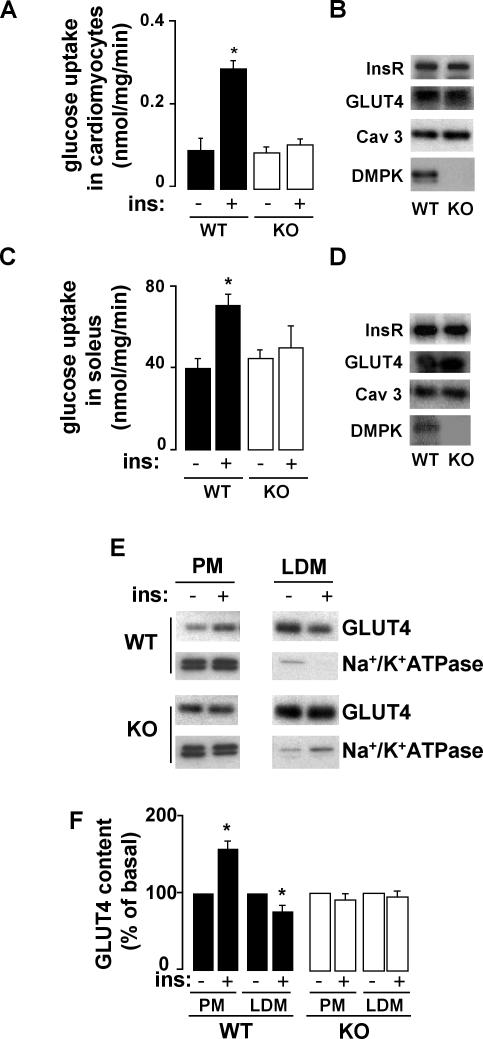
Decreased insulin-dependent glucose transport and abnormal GLUT4 translocation in *dmpk* ^−/−^ muscle. (A, C) *Ex vivo* glucose uptake in myocytes isolated from left ventricles (n = 6 per group) and soleus muscles (n = 3 per group), stimulated with or without insulin (100 nM). (B, D) Insulin receptor, glucose transporter GLUT4, caveolin 3 and DMPK protein contents were analyzed in cardiomyocytes and soleus, respectively, from wild-type (WT) and *dmpk* ^−/−^ (KO) mice (n = 3). (E, F) Subcellular fractions were prepared from cardiac ventricles of basal and insulin-treated mice. (E) Plasma membranes (PM) and low density microsomes (LDM) were analysed by SDS-Page and Western blot. Representative autoradiograms from 3 experiments are shown. Equal amounts of membrane proteins (4 µg) from the different fractions were laid on gels. (F) GLUT4 translocation was quantified from 3 independent experiments. (Data are mean±SEM; *P<0.05 vs unstimulated values)

To examine whether *dmpk* ^−/−^ mice display muscle insulin resistance, we first measured insulin-stimulated glucose transport in cardiac and skeletal muscle. In myocytes isolated from left ventricles and soleus muscles from *dmpk* ^−/−^ mice, insulin-stimulated glucose transport was decreased compared with wild-type mice (Fig. 2 A and C, respectively). The alterations in glucose uptake were not due to a decrease in the insulin receptor or the glucose transporter GLUT4 expression levels (Fig. 2 B and D). Moreover, expression of caveolin 3, a specific muscular marker of *caveolae* that is altered in other forms of muscular dystrophies [24] and is required for insulin-stimulated glucose uptake [25], was also normal in cardiac and skeletal muscles from *dmpk* ^−/−^ mice. We further studied the role of DMPK in muscle insulin sensitivity by analyzing GLUT4 translocation, a critical muscle response to insulin. We performed subcellular fractionations of cardiac muscle membranes from wild-type and *dmpk* ^−/−^ mice. Endosomes and sarcolemmal membrane fractions were separated by successive spins of homogenates (Fig. 2 E). Low density microsome (LDM) fractions were enriched in GLUT4 and contained very low levels of Na^+^/K^+^-ATPase, indicating that such fractions were mostly devoid of plasma membrane. In wild-type mice, GLUT4 content in plasma membrane (PM) fraction was increased 51±15% by insulin injection, at the expense of the LDM pool of GLUT4, which showed 23±4% reduction upon insulin treatment. Insulin-induced translocation of GLUT4 from LDM to PM was not detected in *dmpk* ^−/−^ mice (Fig. 2 F).

**Figure 3:**
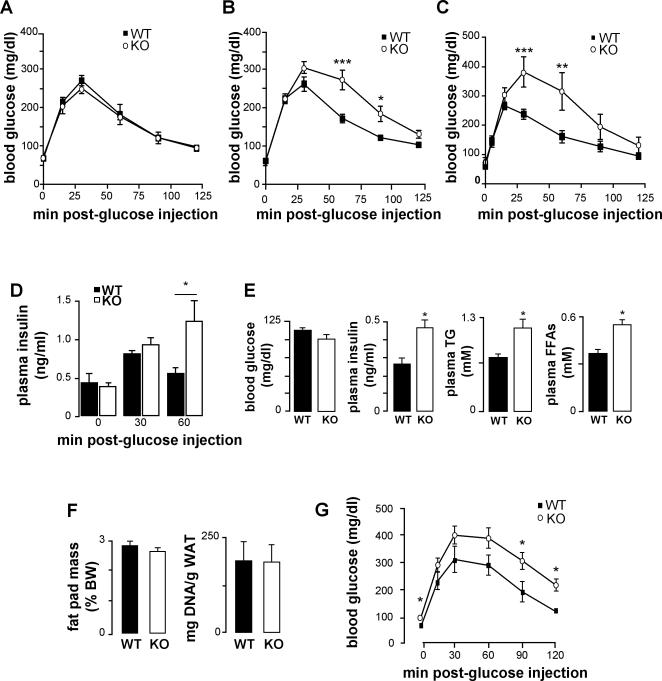
Metabolic parameters in *dmpk* ^−/−^ mice. Glucose tolerance tests of (A) 4-week-old males; (B) 8–10-week-old males; (C) 8–10 week-old females. (wild type , black squares; *dmpk^−/−^* mice, white circles). (n = 5–8 mice per group) (D) Plasma insulin, during glucose tolerance test performed on 16 h-fasted mice (n = 5−8 for each group). (E) Insulin, triglycerides, and free fatty acids (FFAs) concentrations in plasma from fed mice (n = 8–14 for each group). (F) Glucose tolerance tests on 4-month-old males on high fat diet (n = 8 per group; wild type, black squares; *dmpk^−/−^* mice, white circles). Data are mean±SEM; *P<0.05; **P<0.001; ***P<0.0001 vs wild-type values.

Glucose tolerance tests performed at 4 weeks of age were similar for *dmpk* ^−/−^ and wild-type mice (Fig. 3 A). In contrast, at 8–10 weeks of age glucose tolerance in both male and female *dmpk* ^−/−^ mice was altered (Fig. 3 B and C, respectively). Plasma insulin levels measured during glucose tolerance tests were also elevated in *dmpk* ^−/−^ as compared to wild-type mice (Fig. 3 D). However, *dmpk* ^−/−^ mice showed normal fasting glucose and glycemia returned to baseline 2 h after glucose injection in both groups. Fasted *dmpk* ^−/− ^mice showed normal insulin, triglyceride and free fatty acid levels (Table 1) while these parameters in fed animals were higher than those from control animals (Fig. 3 E). No increases in fat-cell mass or fat-cell number were detected in *dmpk* ^−/−^ mice (Fig. 3 F). The glucose intolerant response of *dmpk* ^−/−^ mice placed on a high-fat diet for 8 weeks was more severe than that of *dmpk* ^−/−^ mice on a standard chow diet. Indeed, this metabolic stress significantly increased fasting blood glucose levels as well as glycemia 2 h after the glucose overload in *dmpk* ^−/−^ compared to wild type mice (Fig. 3 G).

**Table 1:** Metabolic parameters in fasted *dmpk*−/− mice.

	*dmpk* +/+	*dmpk* −/−
Weight (g)	18±1	17±2
Blood glucose (mg/dl)	53.33± 4.84	54.60±2.16
Plasma insulin (ng/ml)	0.34±0.08	0.37±0.09
Plasma triglycerides (mM)	0.85±0.06	0.79±0.05

Data are means±SEM, ( n = 8-20)

### DMPK is required for insulin receptor targeting to the plasma membrane

**Figure 4:**
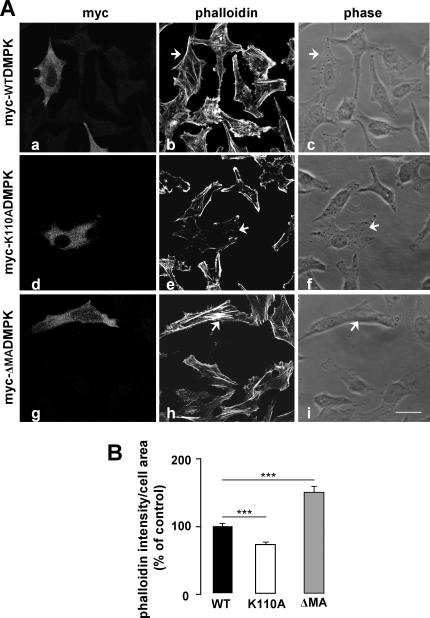
Overexpression of DMPK mutants alters stress fiber formation in HeLa cells. (A) Stress fiber formation was analyzed by phalloidin labeling. Cells were transfected with myc-WTDMPK (a–c), the kinase-dead myc-K110ADMPK mutant (d–f), and the C-terminal lacking myc-ΔMADMPK mutant (g–i). Arrowheads indicate the transfected cells. Scale bar, 24 µm (applies to all panels). Representative images from 3 independent experiments are shown. (B) Stress fiber formation was quantified using the Image J software as phalloidin intensity/cell area. Values for untransfected cells were set as 100%. Means from three independent experiments with 20 cells analyzed per condition are shown. Data are mean±SEM; ***P<0.0001 vs unstransfected cells.

**Figure 5:**
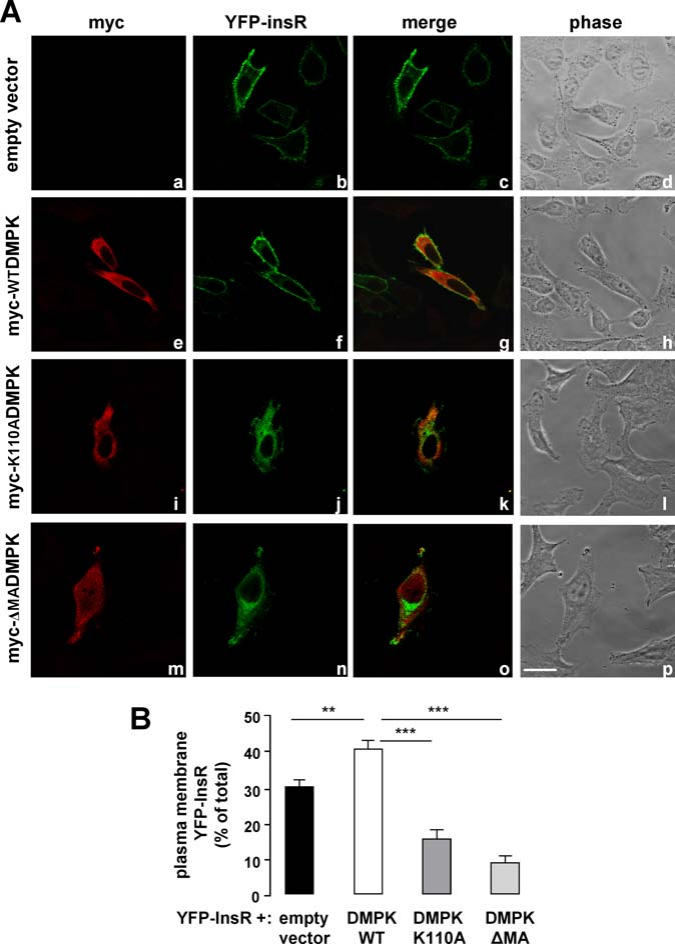
DMPK is required for insulin receptor targeting to the plasma membrane. (A) HeLa cells were transiently co-transfected with YFP-InsR along with control empty vector (a–d), myc-WTDMPK (e–h), myc-K110ADMPK (i–l) or myc-ΔMADMPK (m–p). Shown are representative images of 3 independent experiments. Scale bar, 24 µm (applies to all panels). (B) Receptor percentage at the cell surface was quantified by using Image J software. Means from three independent experiments with 20 cells analyzed per condition are shown. Data are mean±SEM ; **P<0.001; ***P<0.0001 vs unstransfected cells.

**Figure 6:**
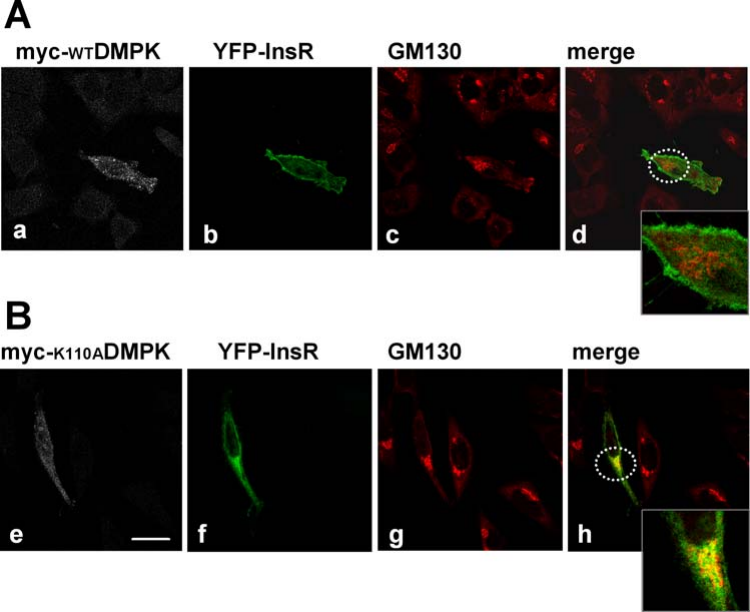
Effect of kinase-deficient mutant DMPK (myc-K110ADMPK) on YFP-InsR subcellular distribution. HeLa cells were transiently co-transfected with YFP-InsR along with myc-WTDMPK (upper panels) or myc-K110ADMPK (lower panels). Cellular distribution of YFP-InsR partially coincides with that of Golgi complex protein GM130 in cells expressing myc-K110ADMPK (panel h). Dashed circles in b and f show the magnified areas. Scale bar, 24 µm. Representative images of 3 independent experiments are shown.

Regarding the molecular mechanism whereby DMPK activity could influence insulin receptor signaling, previous observations led us to analyze the role of DMPK in stress fiber formation: (i) DMPK function has been associated with the regulation of cytoskeleton in lens cells [26], and (ii) it has recently been shown that disruption of the actin cytoskeleton leads to alterations in insulin receptor localization and signaling [27]. We used two mutants of DMPK: myc-K110ADMPK, a kinase deficient form mutated at the ATP-binding site [28], and myc-ΔMADMPK, which lacks C-terminal residues 550-629 but retains kinase activity [22]. HeLa cells were transiently transfected with wild-type myc-DMPK, myc-K110ADMPK or myc-ΔMADMPK. Transfected cells were analyzed after 3-h starvation to determine the stress fiber content in steady state (Fig. 4 A and B). The stress fiber pattern in cells expressing myc-WTDMPK was indistinguishable from that of the surrounding untransfected cells (Fig. 4 A, a–c and Fig. 4 B). Expression of the kinase-dead mutant myc-K110ADMPK resulted in a disassembly of stress fibers (Fig. 4 A, d–f and Fig. 4 B) while myc-ΔMADMPK induced a gross condensation of actin filaments within the cell (Fig. 4 A, g–i and Fig. 4 B). These results were consistent with those previously reported for DMPK in lens cells and for the DMPK homolog ROKα in HeLa cells [26], [29]. Having verified the effect of DMPK mutants in stress fiber formation in HeLa cells, we analyzed their effect in the intracellular trafficking of the insulin receptor. HeLa cells were transiently co-transfected with yellow fluorescent protein-tagged InsR (YFP-InsR) along with myc-WTDMPK, myc-K110ADMPK, myc-ΔMADMPK or empty vector as control. Co-transfected cells were analyzed by confocal immunofluorescence after 3-h starvation to determine the localization of YFP-InsR in steady state. In cells expressing either empty vector or myc-WTDMPK, YFP-InsR was targeted to the cell surface (Fig. 5 A, a–d and e–h, respectively). Quantification analysis showed that the overexpression of myc-WTDMPK induced a significant increase in the receptor density at the plasma membrane compared to control cells (Fig. 5 B). In contrast, in the presence of either myc-K110ADMPK or myc-ΔMADMPK mutants, YFP-InsR was retained in intracellular structures (Fig. 5 A, i–l and m–p, respectively), with no evident co-localization with the DMPK mutants (Fig. 5 A, k and o). For both mutants, the percentages of YFP-InsR at the plasma membrane were significantly decreased compared to control cells (Fig. 5 B). To identify the intracellular compartments in which the YFP-InsR was retained, HeLa cells were transfected with the vector encoding this protein and myc-WTDMPK or myc-K110ADMPK and subjected to staining with anti-myc antibody and a series of markers for different organelles. Co-localization studies revealed that in the presence of myc-K110ADMPK, the intracellularly retained receptor partially coincides with the Golgi matrix protein GM130 (Fig. 6). No evidence of co-localization was detected with early endosomes (EEA1) or to recycling endosomes (transferrin receptor) (not shown).

**Figure 7:**
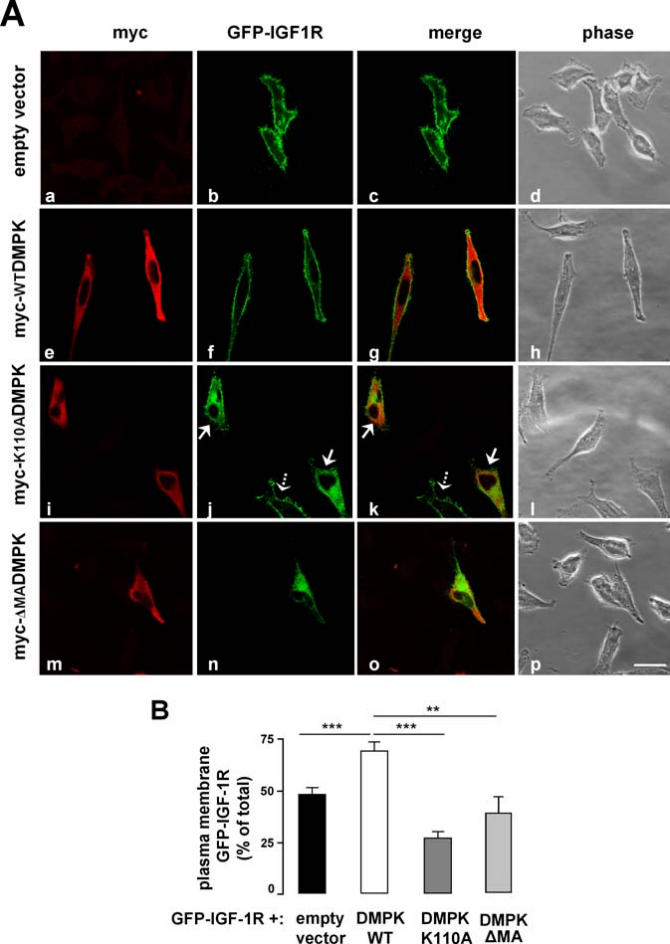
DMPK is required for IGF-1 receptor targeting to the plasma membrane. (A) HeLa cells were transiently co-transfected with GFP-IGF-1 along with control empty vector (a–d), myc-WTDMPK (e–h), myc-K110ADMPK (i–l), or myc-ΔMADMPK- (m–p). Dotted arrows in panel k and l show a cell which only incorporated the GFP-IGF-1 plasmid, expressing the receptor at the plasma membrane. In the same panels, neighboring cells co-expressing the receptor and myc-K110ADMPK are indicated by continuous arrows; in these cells, the accumulation of the receptor in intracellular compartments is observed. Shown are representative images of 3 independent experiments. Scale bar, 24 µm (applies to all panels). (B) Receptor percentage at the cell surface was quantified by using Image J software. Means from three independent experiments with 20 cells analyzed per condition are shown. Data are mean±SEM; **P<0.001; ***P<0.0001 vs unstransfected cells.

To further analyze DMPK role in insulin receptor trafficking, we measured the insulin binding activity in cardiomyocytes isolated from *dmpk* ^−/−^ and wild type mice. ^125^I-insulin binding assays were carried out at 12°C on cells from 16-h fasted mice to analyze cell surface binding in the absence of internalization. Cells from *dmpk* ^−/−^ mice showed a 36±8% (n = 4, p<0.05) decrease in ^125^I-insulin binding at 0.033 nM insulin concentration compared to controls (Table 2). This decrease was associated with a reduced number of receptors at the cell surface with no apparent changes in receptor affinity for the ligand as the percentage of ^125^I-insulin displacement at saturating unlabeled insulin concentrations was similar in both groups (58±13% and 62±8% displacement for wild type and *dmpk* ^−/−^ mice, respectively). To better understand the metabolic phenotype of *dmpk* ^−/−^ mice, we also analyzed the impact of DMPK in the IGF1-receptor intracellular trafficking. The results obtained by co-transfection of GFP-tagged IGF-1 receptor along with myc-WTDMPK, myc-K110ADMPK, myc-ΔMADMPK or empty vector as control were similar to those obtained for the insulin receptor (Fig. 7 A and B).

**Table 2:** Insulin binding to isolated cardiomyocytes.

[insulin] nM	% insulin bound/10^5^ cells	
	*dmpk* ^+/+^	*dmpk* ^−/−^
0.033	6.0±0.5	3.8±0.4*
1	2.4±0.5	1.4±0.3

Results expressed as % of ^125^I-insulin specifically

bound at the indicated insulin concentration.

* *P*<0.05 *vs.* WT (n = 4)

## Discussion

In this study, we addressed the molecular and metabolic function of DMPK, a poorly characterized serine/threonine protein kinase. Consistent with the preferential expression of DMPK in muscle tissues [18], we show that *dmpk* ^−/−^ mice exhibit insulin signaling defects in cardiac and skeletal muscle. Our data indicate that *dmpk* ^−/−^ mice present a significant degree of metabolic alteration, reflected in elevated glucose levels in glucose tolerance tests and increased circulating fed insulin and lipid levels. As observed in DM1 patients [4], *dmpk* ^−/−^ mice show higher concentrations of plasma insulin than wild-type mice in the glucose tolerance tests. These mice exhibit impaired glucose uptake and GLUT4 translocation indicating that decreased insulin sensitivity in muscle could be at the basis of the observed metabolic alterations. Indeed, *dmpk* ^−/− ^mice show normal adiposity and insulin signaling in adipose tissue and liver, in which DMPK is not expressed.

Insulin-induced autophosphorylation of insulin/IGF1-R in response to insulin is influenced by the number of receptors at the cell surface. Correct intracellular trafficking of the InsR is critical for insulin sensitivity and it has been shown that mutations in the InsR gene that impair the transport of the receptor to the plasma membrane lead to type 2 diabetes in humans [30]. After insulin binding, InsR is rapidly internalized and either sent to lysosomes for degradation, or recycled to the plasma membrane for another round of binding, activation, and internalization [31]. Our results show that: (i) the insulin receptor expression levels in *dmpk* ^−/−^ mice are normal; (ii) the insulin binding to the plasma membrane of *dmpk* ^−/−^ cardiomyocytes is decreased; (iii) the overexpression of kinase-deficient and C-terminal truncated DMPK mutants leads to the retention of the InsR in intracellular compartments; and (iv) the overexpression of wild-type myc-DMPK increased the percentage of YFP-InsR at the cell surface. Altogether, these data indicate that DMPK is involved in InsR intracellular trafficking. However, this molecular mechanism cannot fully explain the metabolic alterations observed in *dmpk* ^−/−^ mice, especially considering the previous characterization of the muscle-insulin receptor knockout (MIRKO) mice which do not show glucose intolerance [32]. For this reason, we analyzed whether DMPK also regulated the targeting of IGF-1 receptor to the plasma membrane. Indeed, functional inactivation of the IGF-1 and insulin receptors in skeletal muscle (MKR mice) leads to type 2 diabetes phenotype [33]. Interestingly, the glucose tolerance tests performed in 4-week-old MKR mice are very similar to those observed in 8–10-week-old *dmpk* ^−/−^ animals. However, in contrast to MKR mice, older *dmpk* ^−/−^ mice do not develop type 2 diabetes. The milder phenotype of *dmpk* ^−/−^ mice compared to MKR mice is consistent with a mechanism involving a reduction of InsR and IGF-1R function rather than full inactivation of these receptors.

We show that the kinase and C-terminal domains in DMPK are positive and negative regulators, respectively, of the cytoskeleton reorganization. Similar functions were previously found for DMPK in lens cells [26] and for homologous domains in Rho kinase α [29]. Both kinase and C-terminal domain mutants of DMPK alter insulin and IGF-1 receptor targeting to the plasma membrane and stress-fiber formation; however, whether these effects are functionally related remains to be determined. One possibility is that DMPK could be involved in the biogenesis of Golgi-derived transport carriers through regulation of actin cytoskeleton dynamics. Indeed, when actin dynamics is impaired by a variety of actin toxins that depolymerize or stabilize actin filaments, the Golgi complex shows significant structural changes [34]. Interestingly, we detected that the receptor intracellular accumulation caused by DMPK kinase-deficient mutant partially co-localizes with the Golgi matrix protein GM130.

In summary, our study provides *in vivo* and *in vitro* evidence for the role of DMPK in the regulation of insulin action and glucose homeostasis. Taken together, these findings indicate that reduced DMPK expression may directly influence the onset of insulin-resistance in myotonic dystrophy 1 patients and suggest that DMPK could represent a susceptibility gene to type 2-diabetes.

## Materials and Methods

### Mouse experiments

All animal studies were performed in accordance with the guidelines and under approval of the Institutional Review Committee for the Animal Care and Use of the University of Barcelona and by the animal welfare regulations of the University of California, San Diego. The *dmpk* ^−/+^ heterozygous mice on 129SV background were generated by Reddy *et al.* [13]. These mice were mated to produce littermates that were homozygous for intact DMPK allele (WT) and homozygous for the null DMPK allele (KO). Except when indicated, 3-month-old male mice were used in experiments shown. Mice were backcrossed for at least 9 times maintaining the animals as congenic in the colony. Female mice were also analyzed in most of the studies presented with similar results.

### Physiological assays

The following measurements were performed on randomly fed or on 16-h fasted animals when indicated. We measured blood glucose levels on whole venous blood using an automatic glucose monitor (One Touch Basic, Lifescan). Plasma insulin levels were measured by ELISA, using rat insulin as a standard (Crystal Chem). Free fatty acids and triglyceride levels were quantified from plasma using kits from Wako (NEFA-C Ki and Triglyceride L-Type, respectively). We performed glucose tolerance test on 16 h fasted mice injected intraperitoneally with D-glucose (2 g/kg body weight, Sigma). For high-fat diet treatment, mice were individually placed on a fat-adjusted diet (60 kcal% from fat, Research Diets, Inc., New Brunswick, NJ) for 8 weeks.

### Cardiomyocyte isolation and glucose uptake assays

Left ventricle myocytes from 3-month-old mice were prepared by collagenase digestion as described previously [35] except that cells were finally resuspended in D-glucose-free DMEM (Gibco) supplemented with sodium pyruvate (0.22 mg/ml) and 0.2% BSA. Cardiomyocyte suspensions at 10% cytocrit were incubated with or without 100 nM insulin at 35°C for 30 min. The transport assay was initiated by the addition of 2-deoxy-D-glucose (1 mM final concentration, containing 0.5 µCi of 2-deoxy-D-[^3^H]glucose, Amersham Pharmacia Biotech). Glucose uptake was terminated after 20 min by transferring the cell suspension to microfuge tubes and immediately centrifuged at 100×*g* for 30 s. Cell pellets were washed 3 times in ice-cold 50 mM D-glucose in PBS. Background activity was determined by measuring the transport in a solution that contained 50 mM D-glucose. Cells were lysed with 1 ml of ice-cold 0.1 N NaOH with 0.1% SDS and aliquots were taken for determination of radioactivity and protein levels.

For skeletal muscle glucose uptake assays, mice were sacrificed by cervical dislocation and soleus muscles were rapidly dissected. Muscles were then allowed to recover for 15 min in flasks containing 2 ml of incubation medium (D-glucose-free DMEM (Gibco) supplemented with 0.22 mg/ml sodium pyruvate and 0.2% BSA), continuously oxygenated with 95% O_2_, 5% CO_2_ in a shaking water bath (35°C). After recovery, glucose transport was performed by adding 2-deoxy-D-[^3^H]glucose (1 mM, 1 µCi/ml) and (^14^C)mannitol (19 mM, 0.3 µCi/ml) for 20 min and then, muscles were frozen, weighed, and digested in 1 ml of 0.5 N NaOH. Radioactivity was determined by liquid scintillation counting for dual labels and the extracellular and intracellular spaces were calculated as described [36].

### Subcellular membrane fractionation

Separation of cardiac sarcolemmal and endosomal membranes from mouse hearts was performed by differential centrifugation as previously described [37]. Successive spins at 100×*g,* 5,000×*g,* 20,000×*g,* 50,000×*g* and 100,000×*g* were performed. Membranes pelleting at 20,000×*g* [plasma membrane fraction, PM] were enriched in the plasma membrane marker Na^+^/K^+^-ATPase. To obtain a fraction containing the intracellular GLUT4 pool, the 20,000 *g* supernatant was centrifuged for 30 min at 50,000×*g*, resulting in the separation of a high-density microsome fraction [highly contaminated with plasma membranes] and a low-density microsomes (LDM) fraction in the supernatant. This supernatant was finally ultracentrifuged for 60 min at 100,000×*g* to obtain the LDM pellet. Combining the quantitated signals from LDM and PM fractions indicates that 83±5% of Na^+^/K^+^ ATPase is found in PM fraction, while 77±2% of GLUT4 is in LDM (n = 3, p<0.05).

### ^125^I-insulin binding assays

Insulin binding in suspended cells was measured as described [38]. Human biosynthetic insulin was a kind gift from Eli Lilly Co. (Indianapolis, IN). A^14^-^125^I-human insulin was purchased from Perkin-Elmer (Boston, MA). Cells were exposed to a tracer concentration of A^14^-^125^I-labeled human insulin and varying concentrations of unlabeled human insulin for 4 hr at 12°C. Reactions were terminated by layering duplicate aliquots of the binding reaction over dibutyl phthalate in microcentrifuge tubes and centrifuging at 14,000×g for 30 sec. The supernatant was aspirated off and the radioactivity in cell pellets determined. Specific binding was calculated by subtracting non-specific binding measured in the presence of a large excess (1.67 µM) of unlabeled insulin.

### Weight and DNA content of WAT

Perirenal, perigonadal and subcutaneous adipose tissues were carefully dissected and weighed to determine the fat pad weight as percentage of total body weight. To determine fat pad cell number, perirenal adipose tissue was resected, weighed, and immediately frozen in liquid nitrogen. About 50 µg of tissue were homogenized, and genomic DNA was extracted using Quant-iT PicoGreen (Invitrogen) as described by the manufacturer. DNA was measured by fluorimetric method.

### Biochemical analyses

16-h fasted mice were injected intraperitoneally with D-glucose (1 g/kg body weight) and 10 min later, with insulin (5 U/kg body weight). After 10 min, heart, skeletal muscle, adipose tissue and liver were rapidly extracted, freeze clamped in liquid nitrogen and homogenized as described previously [28]. Control tissues from untreated fasted mice were obtained in parallel. Immunoblotting analyses were performed following standard procedures. Antibodies to mouse DMPK were from Zymed Laboratories. Antibodies to insulin receptor β-subunit, caveolin 3 and caveolin 1 were from BD Transduction Laboratories. All other antibodies used were from Cell Signaling Technology. Immunoblots were scanned and signals were quantified using HP Precisionscan Pro and Syngene Gene Tools software. Shown are representative immunoblot data from at least 3 independent experiments which were quantified and expressed as the mean±SEM relative ratio of phosphoprotein to total protein between untreated and insulin-treated mice.

#### Gene transfer by adenovirus vectors

Adenoviruses expressing myc-tagged human DMPK were generated by homologous recombination as described [28]. Subconfluent C2C12 cells were infected for 2 h with 100 plaque-forming units/cell of adenovirus vector encoding either myc-DMPK or green fluorescent protein (GFP) before the addition of a suitable volume of myogenic culture media (DMEM with 5% horse serum and antibiotics). After 2 days, myotubes were treated with or without 100 nM insulin for 30 min at 37°C, harvested, lysed and analyzed by immunoblotting as described above.

#### Cell transfection and confocal microscopy

For cell transfection experiments, HeLa cells on coverslips were transiently transfected using Lipofectamine 2000 (Invitrogen) according to the manufacturer's instructions. Plasmids used were: myc-tagged wtDMPK and myc-tagged ΔMADMPK [22]; myc-tagged K110ADMPK [28]; yellow fluorescent protein-tagged insulin receptor (YFP-InsR) [39]; and green fluorescent protein-tagged IGF-1 receptor (GFP-IGF-1) [40]. Thirty-six hours after transfection, cells were serum starved for 3 h, then washed in PBS, fixed for 30 minutes in 4% w/v paraformaldehyde and processed for immunofluorescence as described above. Antibodies used were anti-myc rabbit polyclonal antibody (Upstate), anti-myc mAb 9E10 (ATCC), mAb anti-GM130 [Amersham Biosciences], anti-mouse Cy5-conjugated IgG (Jackson), anti-rabbit Alexa Fluor 555 IgG (Molecular Probes) and anti-mouse Alexa Fluor 594 IgG [Molecular Probes]. For staining of actin filaments, the coverslips were incubated with phalloidin conjugated to Alexa Fluor 594 (Molecular Probes). Cells were examined using a Zeiss LSM510 confocal laser microscope with an oil immersion 63×/NA1.3 objective. Micrographs shown are representative optical sections imaged through the centre of the cell. At least 20 cells for each condition of 3 independent experiments were examined and subjected to quantification analyses using Image J software.

### Statistical analysis

Data are presented as mean±standard error. Statistical analysis was performed using a two-tailed unpaired *t*-test. Two-way analysis of variance (ANOVA) was applied for multiple comparisons, followed by the Bonferroni *post hoc* test. Values of *P*<0.05 were considered as statistically significant.
